# Machine Learning Classification of Smoking Behaviours—From Social Environment to the Prefrontal Cortex

**DOI:** 10.1111/adb.70056

**Published:** 2025-08-06

**Authors:** Pablo Reinhardt, Norman Zacharias, Marinus Fislage, Justin Böhmer, Barbara Hollunder, Zala Reppmann, Anton Wiehe, Rebecca Rajwich, Nanne Dominick, Kerstin Ritter, Malek Bajbouj, Thomas Wienker, Jürgen Gallinat, Norbert Thürauf, Johannes Kornhuber, Falk Kiefer, Michael Wagner, Oliver Tüscher, Henrik Walter, Georg Winterer

**Affiliations:** ^1^ Department of Psychiatry and Psychotherapy Charité – Universitätsmedizin Berlin Berlin Germany; ^2^ Department of Otolaryngology Charité – Universitätsmedizin Berlin Berlin Germany; ^3^ Department of Anesthesiology and Intraoperative Intensive Care Medicine Charité – Universitätsmedizin Berlin Berlin Germany; ^4^ Pharmaimage Biomarker Solutions Inc. Cambridge Massachusetts USA; ^5^ Department of Neurology Charité – Universitätsmedizin Berlin Berlin Germany; ^6^ Einstein Center for Neurosciences Berlin Charité – Universitätsmedizin Berlin Berlin Germany; ^7^ Berlin School of Mind and Brain Humboldt‐Universität zu Berlin Berlin Germany; ^8^ Hertie Institute for AI in Brain Health University of Tübingen Germany; ^9^ Department of Molecular Human Genetics Max Planck Institute for Molecular Genetics Berlin Germany; ^10^ Department of Psychiatry University Hospital Hamburg Hamburg Germany; ^11^ Department of Psychiatry and Psychotherapy University Clinic, Friedrich‐Alexander‐University of Erlangen‐Nuremberg Erlangen Germany; ^12^ Department of Addictive Behaviour and Addiction Medicine, Central Institute of Mental Health, Medical Faculty Mannheim Heidelberg University Mannheim Germany; ^13^ Department of Psychiatry University Hospital Bonn Bonn Germany; ^14^ Department of Psychiatry University Hospital Mainz Mainz Germany

**Keywords:** classification, prefrontal function, tobacco use behaviour

## Abstract

The pronounced heterogeneity in smoking trajectories—ranging from occasional or heavy use to successful quitting —highlights substantial interindividual variation within the smoking population. Machine learning is particularly well suited to capture these complex patterns that may be challenging for traditional inferential statistics to uncover. In this study, we applied machine learning to data from a population‐based cohort to identify multimodal markers that distinguish smokers from never smokers at baseline and predict long‐term cessation success at a 10‐year follow‐up. We employed 10 times repeated nested cross‐validation (10 outer folds, 5 inner folds) to analyse baseline data (T1) from 707 smokers—including 222 heavy smokers (FTND ≥ 4)—and 864 never smokers for smoking status classification. At the 10‐year follow‐up (T2), we further classified 60 successful quitters (≥ 1 year abstinent) versus 81 non‐quitters. Feature importance was assessed using averaged SHAP values derived from test set predictions. Classification models achieved the following performance, expressed by the area under the receiver operating characteristic curve (AUROC; mean ± SD): smokers versus never smokers, 0.85 ± 0.03; heavy smokers versus never smokers, 0.92 ± 0.03; and quitters versus non‐quitters, 0.68 ± 0.13. SHAP analysis identified markers of frontal functioning, cognitive control and smoking behaviour within the social environment among the most influential predictors of both smoking status and cessation success. In conclusion, our machine learning analyses support a multifactorial model of smoking behaviour and cessation success, which may inform nuanced risk stratification to advance the development of personalized cessation strategies.

## Graphical abstract

Applying machine learning to a 10‐year longitudinal cohort, this study identifies stable predictors of smoking status and cessation. By integrating multimodal data – including electrophysiological and neuropsychological measures – our models achieved high classification accuracy and retained meaningful predictive power, prospectively distinguishing quitters from persistent smokers a decade later. Frontal brain function, cognitive control and social smoking environments emerged as the most salient features, highlighting robust markers that may inform personalized cessation strategies.

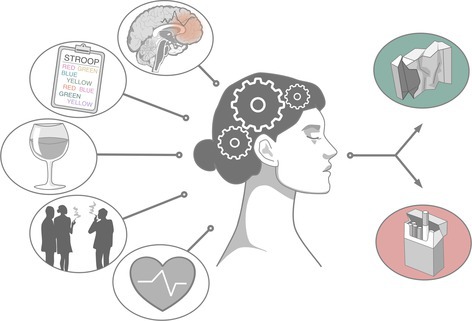

## 1 Introduction

Tobacco addiction, responsible for over 13% of global fatalities, remains a leading cause of ill health and premature death [1]. A nuanced understanding of the factors underlying tobacco use and cessation is essential for advancing effective prevention and treatment strategies. Yet, despite extensive research, the considerable interindividual variability in smoking behaviour remains largely elusive [2]. Namely, individuals present with marked differences in their smoking trajectories—from never smoking in the first place, to different degrees of tobacco consumption, or its successful cessation. These interindividual differences in the behavioural patterns of tobacco use likely stem from a complex interplay of sociodemographic, environmental, neuropsychological and neurobiological factors [3].

Specifically, research has consistently highlighted the pivotal role of the prefrontal cortex (PFC) in chronic addiction and abstinence [4, 5, 6], as corroborated by causal brain mapping studies investigating connectivity from lesions [7] or targeted stimulation sites [8] related to tobacco use behaviour. In line with this, reduced cognitive control and increased impulsivity [9, 10, 11], reflected in the form of functional alterations within the PFC, are considered a hallmark dysfunction underlying substance use disorders. For instance, impaired prefrontal inhibition in chronic smokers coincided with reduced electrophysiological P50 frontal gamma oscillations derived from sensory gating, a passive listening task utilized to map early processes of selective attention engaged in inhibition [12].

In addition to neurobiological and neuropsychological markers, social and environmental factors—especially peer influence and sociodemographic variables such as urbanicity and employment status—have been recognized as significant determinants of both smoking initiation and cessation success [13, 14]. For instance, smoking behaviour in participants' social environment considerably impacted the inclination to smoke [15] as well as successful cessation attempts [14].

While the significance of these multifaceted factors is well acknowledged, our understanding of which variables most effectively predict smoking behaviours and cessation success remains limited [15]. Mechanistic studies have advanced insights into the biology of addiction; however, the emerging field of precision psychiatry is shifting focus towards clinical endpoint classifications. By leveraging machine learning, it aims to forecast individual outcomes across heterogeneous data sources—for instance, predicting the responses of psychiatric patients to medication [16, 17] or their likelihood of treatment success [18, 19].

Despite growing interest in data‐driven approaches, few studies on tobacco use disorder have applied machine learning to both cross‐sectional and longitudinal data to identify phenotypic variables—spanning a wide range of domains—that most robustly predict smoking behaviour and cessation outcomes. Leveraging data from a nationally representative cohort on nicotine dependence [20] and a follow‐up online assessment 10 years later, herein, we apply machine learning techniques to classify cross‐sectional and longitudinal smoking‐related phenotypes of (*heavy‐)smokers*, *never smokers* and *(non‐)quitters*, drawing upon a comprehensive range of phenotypic smoking‐related variables. Following this approach, we aim to uncover key predictors of smoking behaviour and long‐term cessation success.

## 2 Methods

### 2.1 Study Design and Population

In this study, we utilize machine learning classification of smoking‐related behaviours on a longitudinal dataset (comprised of baseline and follow‐up), based on two waves of data collection: first, the population‐based Nicotine Cohort Study (NCS) [21] conducted between 2007 and 2009, and, second, a follow‐up online assessment of a subsample of NCS participants, conducted approximately 10 years later (2019–2022). The NCS cohort (*N* = 2396) constitutes *N* = 1116 current regular *smokers* and *N* = 1280 *never smokers* tested on a wide array of phenotypical sociodemographic‐environmental, clinical, neuropsychological, physiological and electrophysiological (EEG) variables [10, 12, 21, 22]. EEG data were collected in the resting state and during an auditory sensory gating paradigm [15], utilizing 32‐channel measurements (details can be found in [12]). Nicotine dependence levels of smokers were assessed utilizing the Fagerström Test for Nicotine Dependence (FTND), with *heavy s*moking defined by FTND scores of four or above. Between 2019 and 2022, *N* = 2000 NCS participants were invited to participate in a follow‐up online assessment collecting variables on sociodemographic information, alcohol and tobacco use, mood and stress. The final sample of follow‐up responders (*N* = 452) comprised *never smokers* (*N* = 311) and former smokers (*N* = 141), with the latter group subdivided into *quitters* (*N* = 60), who had stopped smoking for at least 1 year before follow‐up, and *non‐quitters* (*N* = 81), who had continued smoking.

**FIGURE 1:**
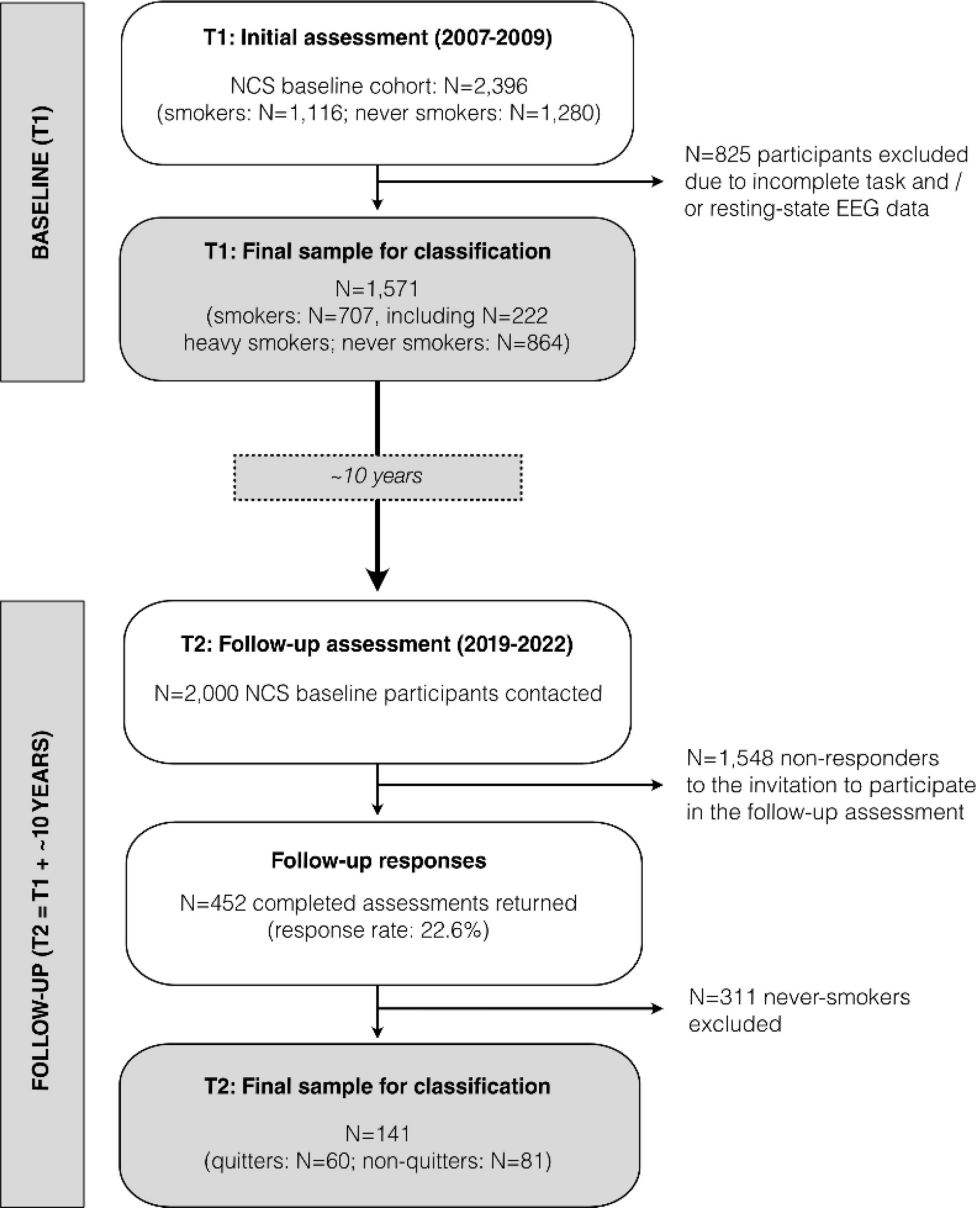
**Study design and sampling characteristics.** The flow diagram depicts the design of the study sample collection, starting from the first wave (T1, top of the flow diagram) to the second wave (T2, bottom of the flow diagram). EEG = electroencephalography; NCS = Nicotine Cohort Study.

The study analysed three main classification models: *smokers* versus *never smokers* and *heavy smokers* versus *never smokers*, leveraging retrospective data from baseline, as well as *quitters* versus *non‐quitters*, leveraging baseline as well as follow‐up data 10 years later. Figure 1 comprises a schematic overview of the samples included.

### 2.2 Sample Selection for Classification Models

To enhance model performance and reliability, we applied tailored inclusion criteria for each classification. For the baseline models—(A) *smokers* versus *never smokers* and (B) *heavy smokers* versus *never smokers*—participants were selected based on the availability of both task‐based and resting‐state EEG data, the study's largest variable domain. For classification model (C) (*quitters* vs. *non‐quitters*), the 10‐year gap between the initial and follow‐up data collection inevitably led to a substantially reduced sample size. Considering the high value of each individual subject and the uniqueness of the study cohort, at the same time, we decided to include all participants who successfully completed the follow‐up online survey—irrespective of EEG data availability at baseline.

For classification models (A) and (B), a total of *N* = 707 *smokers* (mean age: 36.8 ± 13 years; 45% female) and *N* = 864 *never smokers* (mean age: 36.6 ± 13 years; 40% female), comprising *N* = 222 *heavy smokers* (40.4 ± 12 years; 48% female), was included. Classification model (C) was comprised of *N* = 60 *quitters* (35.5 ± 12.3 years; 38% female) and *N* = 81 *non‐quitters* (38.8 ± 11.8 years; 40% female).

### 2.3 Included Features

We included a comprehensive collection of variables from all available domains, encompassing sociodemographic‐environmental information (*N* = 24 variables), clinical questionnaires (*N* = 23 variables), neuropsychological (*N* = 35 variables) and electrophysiological variables (*N* = 126).

For classification (C) of *quitters* versus *non‐quitters*, variables collected at follow‐up, including sociodemographic‐environmental and clinical variables (e.g., alcohol use or stress), were additionally incorporated. Moreover, specific baseline variables pertaining to smoking behaviour (e.g., packyears, pertaining to the number of smoking years multiplied by the number of cigarette pack use per day) were implemented. Table S1 comprises details on the full list of included variables.

The baseline sample of *smokers* and *never smokers* exhibited a total of 1.3% of missing questionnaire values. The follow‐up sample of *quitters* versus *non‐quitters* comprised a total of 18% of missing data points, with 13.1% due to incomplete EEG sensory gating data.

### 2.4 Classification Pipeline and Data Processing

We developed classification pipelines using scikit‐learn [23], imbalanced‐learn [24] and TensorFlow [25] consisting of four sequential steps: (1) feature imputation using mode values, (2) outlier removal via isolation forest, (3) class balancing using SMOTETomek and (4) XGBoost classifier [26] training. For classification (C) (*quitters* vs. *non‐quitters*), we additionally employed the encoder component of an autoencoder trained on baseline EEG data to engineer 12 latent components within each cross‐validation fold (see Table S2 for details on the model architecture and Figure S1 for feature importance).

### 2.5 Model Evaluation and Analysis of Feature Importance

Using nested cross‐validation [27] (10 outer/5 inner folds, 10 repetitions), we tuned models via randomized grid search. Performance was evaluated using AUROC (Table S3 and Figure S2), F1 score, precision, recall and accuracy. For feature importance analysis, we leveraged SHAP (Shapley additive explanations) [28], a model‐agnostic, game‐theoretical approach utilized to derive the relative impact of a feature on the performance of a machine learning model. SHAP values were computed from test predictions for each fold and repetition, aggregated across folds and repetitions, and finally averaged across samples.

### 2.6 Additional Model Evaluation and Robustness Analyses

We also examined whether baseline data alone, in contrast to our primary analysis using longitudinal questionnaire data, could predict smoking status at the 10‐year follow‐up, with consideration of expected model performance constraints (Table S4 and Figure S3).

To ensure the robustness of our classification model (C) (*quitters* vs. *non‐quitters*) based on follow‐up measurements, we conducted additional experiments to address potential concerns. First, we assessed whether imputing missing EEG values—routinely done during preprocessing within cross‐validation—might negatively impact the model's performance and stability, especially in the context of smaller sample sizes. To further evaluate the *quitters* versus *non‐quitters* classification, we conducted a replication using random sub‐sampling with replacement within the repeated nested cross‐validation procedure. This approach reduced the amount of missing EEG data that required imputation per sample (Table S5 and Figure S4).

Moreover, we tested if our findings based on SHAP feature importance could be replicated leveraging predictive modelling approaches beyond XGBoost. Specifically, we repeated classification (C) leveraging random forest and stacking for classification (Table S6 and Figure S5).

## 3 Results

### 3.1 Classification of Cross‐Sectional Smoking‐Related Phenotypes

#### 3.1.1 (A) Smokers Versus Never Smokers

**FIGURE 2:**
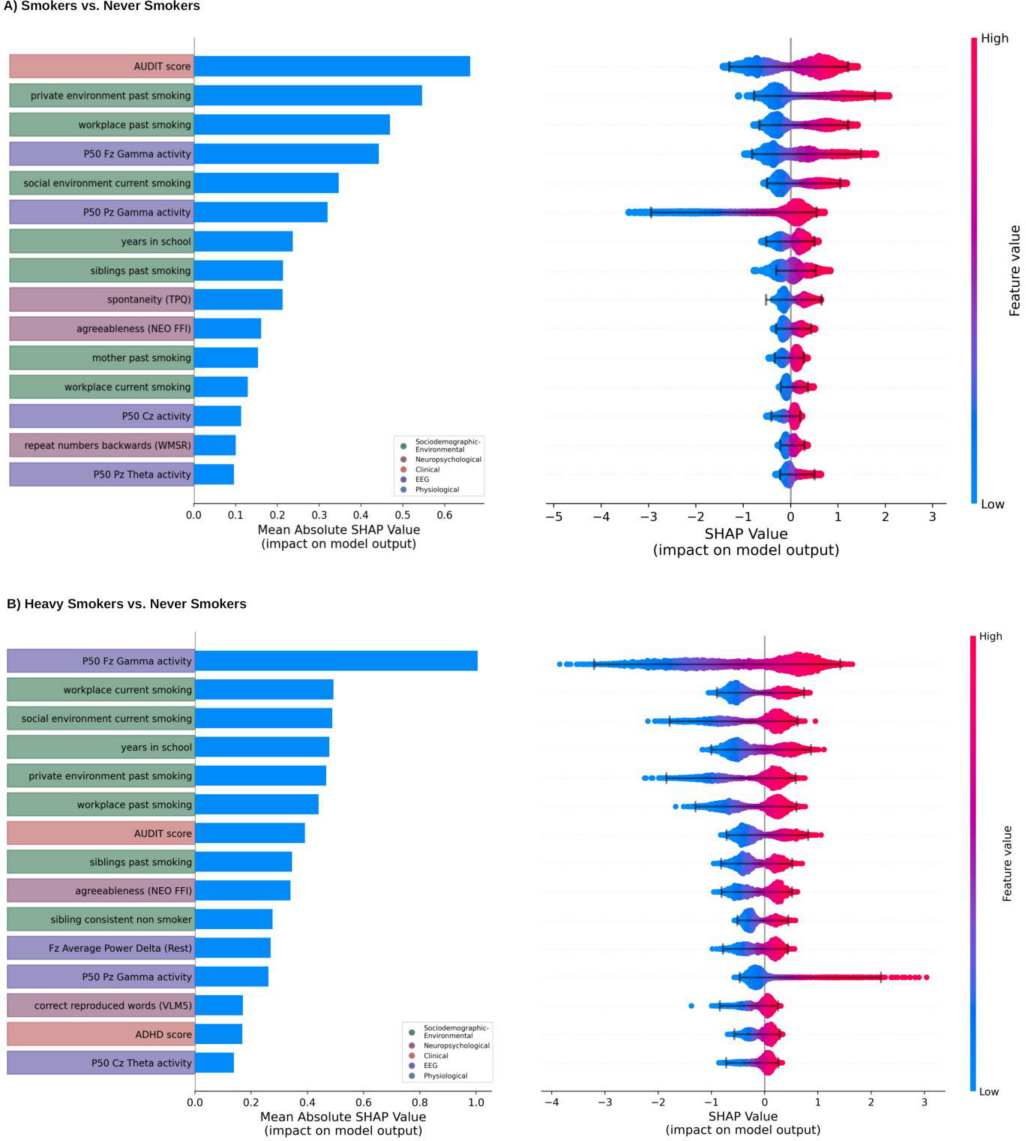
**Shapley (SHAP) values for the 15 most impactful features within baseline classification models, with colour coding based on the feature domain.** (A) *Smokers* (*N* = 707) versus *never smokers* (*N* = 864). (B) *Heavy smokers* (*N* = 222) versus *never smokers* (*N* = 864). *Left:* Bar plots show mean feature impact in descending order. *Right:* Bee swarm plots display individual SHAP values, where positive values indicate contribution to smoker (A) or heavy smoker (B) classification, and negative values to never smoker classification. Absolute SHAP values represent impact magnitude. ADHD = Attention Disorder Hyperactivity Checklist; AUDIT = Alcohol Use Disorders Identification Test; CPT = Continuous Performance Test; EEG = electroencephalography; NEO‐FFI = NEO Five‐Factor Inventory; TPQ = Tridimensional Personality Questionnaire; VLM 5 = Verbal Learning and Memory Test (correct words after delayed recall).

The *smokers* versus *never smokers* classification model achieved the following performance (mean ± SD): weighted f1 0.78 ± 0.03, precision 0.77 ± 0.03, recall 0.77 ± 0.03 and AUROC 0.85 ± 0.03 (Table S3). Key features included AUDIT score, smoking in the social environment and prefrontal (Fz) sensory gating P50 components in the gamma band (Figure 2A). Bee swarm visualization showed that smoking was associated with higher AUDIT scores, increased smoking in private and professional environments, and lower frontal P50 gamma band sensory gating activity.

#### 3.1.2 (B) Heavy Smokers Versus Never Smokers

Classification model (B) (heavy smokers versus never smokers) yielded the following performance (mean ± SD): weighted f1 0.88 ± 0.03, AUROC 0.92 ± 0.03, precision 0.88 ± 0.03 and recall 0.89 ± 0.02 (Table S3). From the electrophysiological domain, the most impactful features included sensory gated prefrontal (as well as parietal) P50 gamma band neural activity and resting state derived prefrontal average power (Figure 2B). From the sociodemographic‐environmental domain, key features were smoking in private and professional environments and siblings' smoking behaviour. The bee swarm visualization indicated that heavy smoking is associated with lower sensory gated prefrontal P50 gamma band neural activity and increased smoking within the social environment (Figure 2B, right).

### 3.2 Longitudinal Classification: (C) Quitters Versus Non‐Quitters (10‐Year Follow‐Up)

**FIGURE 3:**
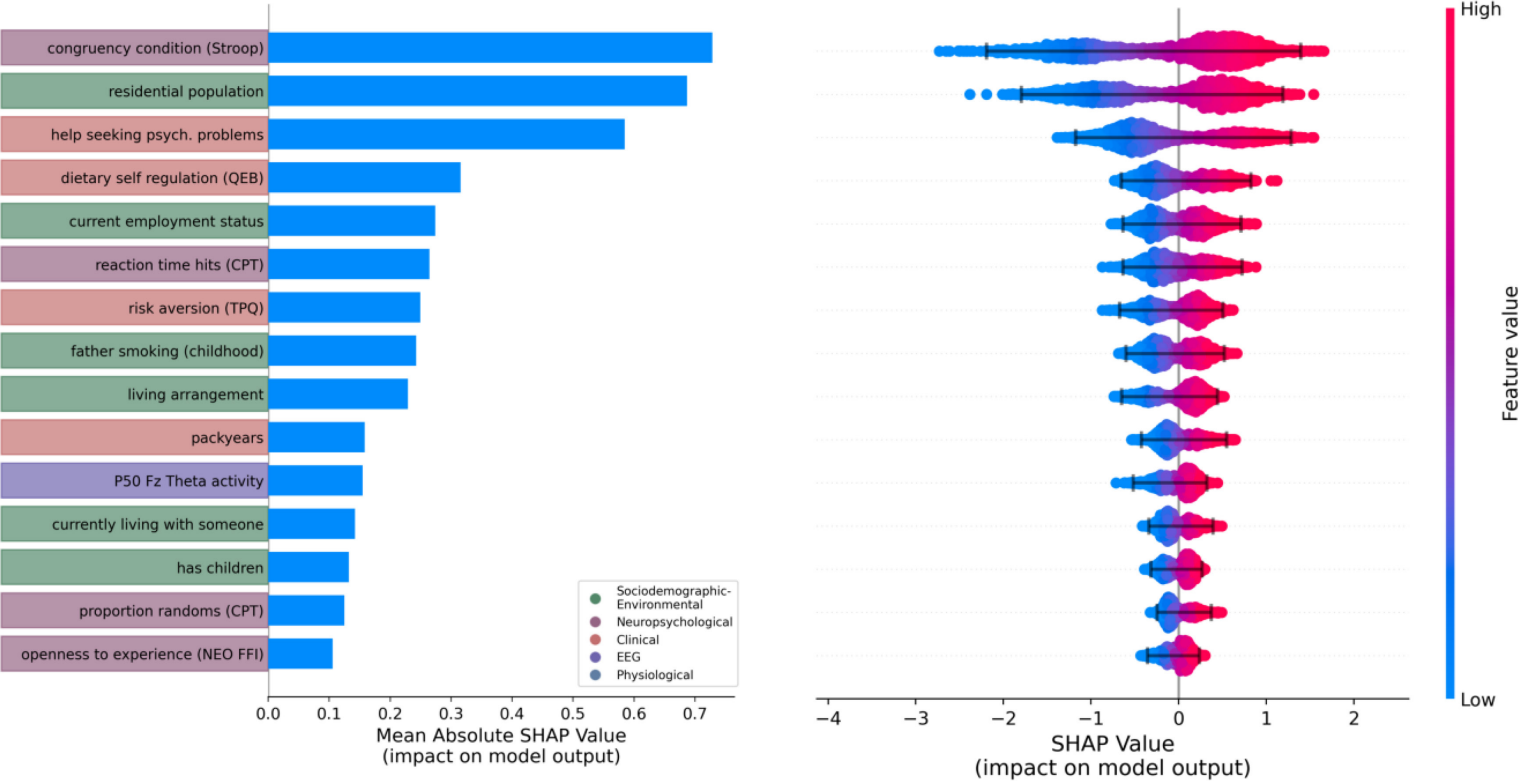
**Shapley (SHAP) values for the 15 most important features in quitters (*N* = 60) versus non‐quitters (*N* = 81) classification, with colour coding based on the feature domain.** Features are ranked by importance. *Left:* Bar plot showing mean feature impact. *Right:* Bee swarm plot displaying individual SHAP values, where positive values indicate contribution to quitter classification and negative values to non‐quitter classification. Absolute SHAP values represent impact magnitude. CPT = Continuous Performance Test; EEG = electroencephalography; TPQ = Tridimensional Personality Questionnaire; NEO‐FFI = NEO Five‐Factor Inventory; Stroop congruent condition = first component of Color‐Word Interference Test.

The *quitters* versus *non‐quitters* classification model achieved the following performance scores (mean ± SD): AUROC 0.68 ± 0.13, weighted f1 0.62 ± 0.13, precision 0.59 ± 0.22 and recall 0.50 ± 0.24 (Table S3). Key predictive features included the Stroop task's congruent condition, resident size, cognitive control of eating behaviour (QEB) and CPT reaction time hits (Figure 3). Bee swarm analysis revealed that successful quitting was associated with higher cognitive control of eating behaviour, increased help‐seeking for psychiatric problems, longer Stroop congruent completion times, fewer pack years and lower risk aversion (TPQ).

### 3.3 Sensitivity Analysis

**FIGURE 4:**
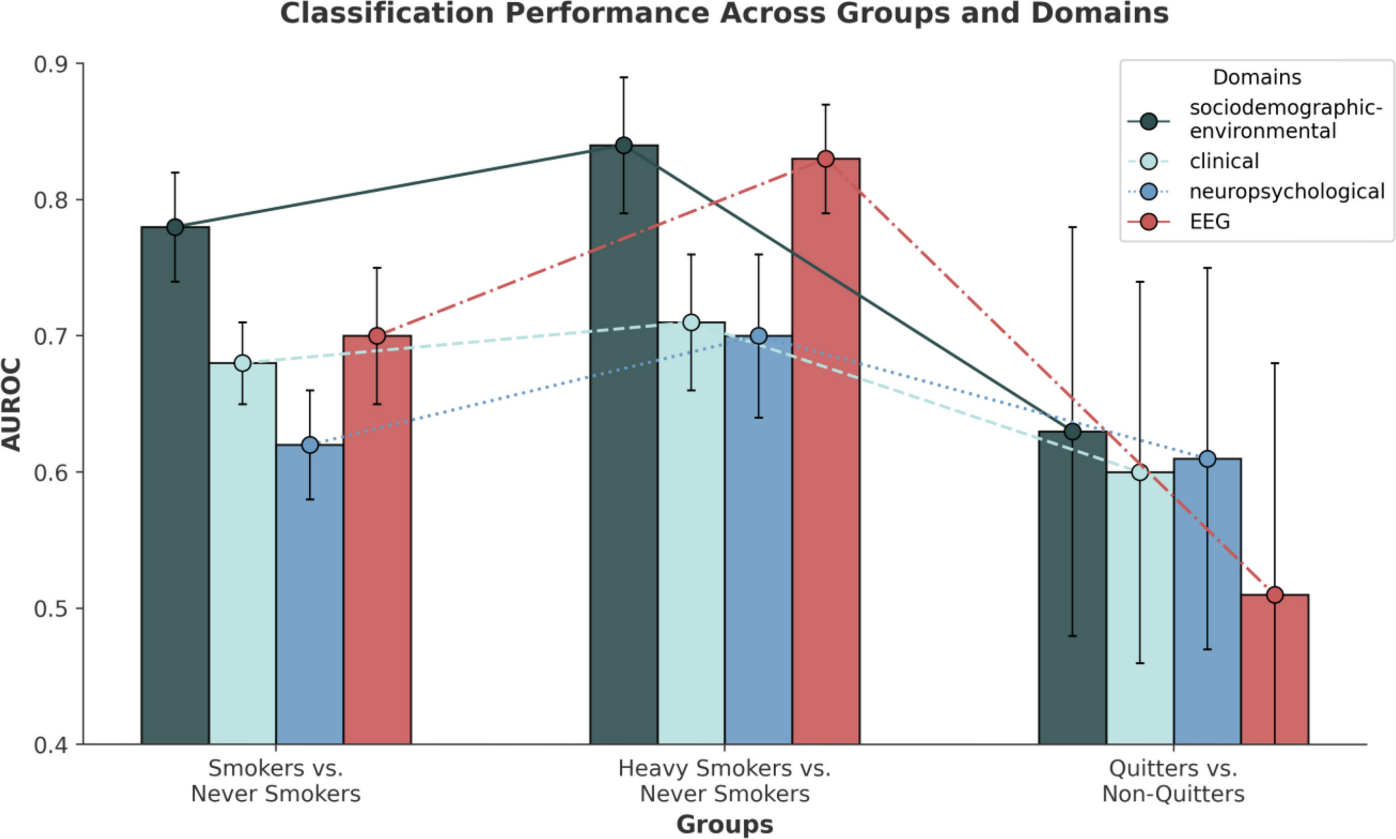
**Sensitivity analysis of model performance by domain for each classification model.** Domain‐specific classification performance for (A) *smokers* versus *never smokers*, (B) *heavy smokers* versus *never smokers* and (C) *quitters* versus *non‐quitters*. Bars represent mean AUROC scores with standard deviations from 10‐fold cross‐validation, computed using a pipeline with imputation, outlier removal and XGBoost classification. The connected points above each bar link the mean AUROC values of each domain across the three group comparisons, allowing to trace how a specific domain's performance varies between different classification tasks. For example, following the red line (EEG features) shows strong performance (AUROC = 0.83) in distinguishing heavy smokers from never smokers, but substantially lower performance (AUROC = 0.51) for the quitters vs. non‐quitters comparison. Error bars on the bars indicate the standard deviation from cross‐validation. AUROC = area under the receiver operating curve; EEG = electroencephalography; STD = standard deviation.

To determine the relative impact of each feature domain, we evaluated the classification performance of models (A)–(C) by using categorically grouped features (feature domains), maintaining identical model parameters (Table S7), visualized in Figure 4. For *smokers* versus *never smokers*, the AUROC scores (mean ± SD) were 0.78 ± 0.04 and 0.7 ± 0.05 for sociodemographic‐environmental and EEG domains, respectively. The *heavy smokers* versus *never smokers* classification achieved comparable scores of 0.84 ± 0.05 (sociodemographic‐environmental) and 0.83 ± 0.04 (EEG). For *quitters* versus *non‐quitters*, the sociodemographic‐environmental domain (0.63 ± 0.14) outperformed the EEG domain (0.51 ± 0.17). While EEG and sociodemographic‐environmental features were key for *smoking* and *quitting* classifications, the distinction between *heavy smokers* and *never smokers* relied more evenly on all domains.

## 4 Discussion

Building on recent advancements highlighting the effective use of multimodal predictors in modelling behavioural trajectories in substance use disorders [29, 30, 31], we demonstrate that integrating EEG, neuropsychological, clinical, sociodemographic‐environmental and physiological measures into a unified machine‐learning framework enables reliable prediction of smoking‐related phenotypes.

Several key conclusions emerge from our findings. First, multimodal classification models incorporating EEG, neuropsychological and sociodemographic‐environmental measures accurately distinguished current smokers—including heavy smokers—from never smokers at baseline, achieving AUROC values between 0.85 and 0.92. Notably, predictive accuracy remained moderate even at 10‐year follow‐up, with models classifying quitters versus persistent smokers reaching an AUROC of 0.68, indicating meaningful temporal stability of key predictive features. Classification performance was primarily driven by frontal P50 gamma activity, cognitive control as measured by the Stroop task (congruent condition) and the smoking behaviour within the participants' social environments. Sensitivity analyses further confirmed electrophysiological and sociodemographic‐environmental measures as the most robust contributors, underscoring the importance of a multi‐domain integration for understanding long‐term trajectories of smoking behaviour.

Recently, machine learning has gained traction in the study of substance use disorders for its ability to uncover patterns in data that were previously difficult to detect with traditional statistical methods. For instance, studies utilizing large datasets like PATH have achieved moderate success (≈ 70%–72% accuracy) in predicting smoking cessation or onset over 1‐year intervals, leveraging machine learning models that incorporated demographic characteristics, smoking behaviours, health conditions and psychosocial factors [28, 29]. Similarly, clinical trial data combining EEG and psychological measures have demonstrated moderate accuracy (concordance index of ≈ 0.68, reflecting the model's ability to accurately rank individuals by their relapse risk) in predicting relapse over a 12‐month duration in adults [21]. Multimodal approaches have also shown promise in predicting outcomes in other substance use disorders, including alcohol use disorder, where the integration of EEG, genetic and clinical data has significantly enhanced prediction accuracy for remission or persistent use compared to single‐modal approaches [31]. Critically, our study extends this multi‐domain classification approach to a much longer follow‐up period—spanning a decade—providing insights into the long‐term trajectory of smoking behaviour.

Although moderate, the follow‐up classification performance observed in our study exceeds what is typically expected over such an extended time frame, suggesting that the identified multi‐domain features reflect relatively stable and predictive characteristics of smoking behaviour over time. While high baseline accuracy highlights a strong association of these features with current smoking status, the sustained predictive power after a decade implies these factors may reflect enduring individual traits or environmental contexts related to smoking, although other variables likely gain influence over such an extended period. The prominence of both electrophysiological and sociodemographic predictors highlights the complex interplay between individual neurobiological predispositions and environmental influences in shaping smoking behaviour. These findings suggest that effective smoking cessation and prevention strategies may be enhanced by targeting both individual‐level cognitive and neural mechanisms, as well as broader sociodemographic‐environmental factors.

Multiple indicators of prefrontal functioning consistently emerged as the most salient predictors for accurately classifying both smoking status (*smokers* vs. *never smokers*) and smoking cessation outcomes. In particular, elevated frontal gamma‐band (P50) activity and performance differences on the Stroop task under congruent conditions—electrophysiological and behavioural markers of fronto‐cortical regulation, respectively—were among the top contributors to model performance. These findings align with a large body of converging evidence from neuroimaging and non‐invasive brain stimulation (NIBS) research, underscoring the central role of prefrontal circuits in the neurobiological mechanisms underlying nicotine addiction [27, 32, 33].

Functional magnetic resonance imaging (fMRI) studies have repeatedly linked deficits in cognitive control and executive functioning among smokers to hypoactivation in key regions of the PFC, most notably the dorsolateral prefrontal cortex (DLPFC) and anterior cingulate cortex (ACC) [34, 35]. Meta‐analyses have further highlighted reduced activation in the left dorsal ACC and right middle frontal gyrus (a component of the DLPFC) during response inhibition tasks in current smokers [34, 36, 37]. Treatment studies reinforce this interpretation. For example, varenicline treatment has been associated with increased task‐related activation in the dorsal ACC, medial frontal cortex and bilateral DLPFC, alongside decreased activation in the rostral ACC, ventromedial PFC (vmPFC) and orbitofrontal cortex (OFC) [38]. These changes suggest that successful cessation may rely on the restoration of top‐down control via enhanced function of dorsal prefrontal regions. In adolescents, greater post‐treatment reductions in Stroop‐related ACC activation have also been linked to longer periods of abstinence, indicating more efficient prefrontal processing supports sustained quitting [11, 39].

Further insight into the key role of prefrontal functioning in substance use disorders, specifically tobacco use disorder, comes from functional neuroimaging studies employing cue‐reactivity, a well‐established predictor of relapse that is characterized by responsivity or craving indexed by exposure to smoking‐related stimuli [8, 40, 41, 42]. Meta‐analyses of fMRI cue‐reactivity studies have demonstrated that exposure to smoking‐related cues elicits increased activity in various PFC regions, including the DLPFC, ACC and OFC [41]. For instance, a meta‐analysis by Engelmann and colleagues [41] revealed reliable activation in the dorsal and medial PFC in response to smoking cues, with the superior frontal gyrus showing more robust activation in deprived smokers. EEG studies support this evidence, with findings indicating that smokers exhibit increased low‐theta coherence in frontal brain regions when exposed to smoking cues, and this activity correlates with self‐reported craving [8, 43, 44]. Notably, both fMRI and EEG findings highlight the PFC's involvement in processing the salience of drug‐related cues and in the executive functions necessary to regulate craving and prevent substance use [41, 42].

Complementing these neuroimaging findings, NIBS studies offer causal evidence for the involvement of specific PFC subregions [7, 45, 46]. Randomized controlled trials have shown that targeted stimulation of the DLPFC using techniques such as repetitive transcranial magnetic stimulation (rTMS) or transcranial direct current stimulation (tDCS) can significantly reduce craving, cigarette consumption and nicotine dependence, while improving abstinence outcomes relative to sham controls [8, 47]. These effects are typically attributed to enhanced top‐down regulation mediated by DLPFC activity—further validating the relevance of the prefrontal circuits implicated by our study's electrophysiological (frontal gamma) and behavioural (Stroop) markers.

Aligning with our findings that underscore the significance of prefrontal neural and cognitive control in classifying smoking phenotypes, SHAP analysis further revealed additional neuropsychological variables of relevance. Notably, our SHAP analysis identified the Continuous Performance Task's (CPT) hit reaction time as an important predictor of smoking behaviour. This is particularly relevant given that CPT performance is commonly interpreted as capturing sustained attention and impulse control—both cognitive domains often found to be compromised in individuals who smoke [48, 49]. The predictive utility of CPT in our model may thus reflect the underlying cognitive deficits of impulsivity and attention deficits, which are conceptually related to, and have been associated in other research with, aspects of sensory processing like P50 gating [50, 51].

Consistent with this, cognitive restraint—assessed via the German Questionnaire on Eating Behaviour (FEV) as an index of dietary control—emerged as a further meaningful predictor. This aligns with broader evidence connecting cognitive control to general self‐regulatory capacity, including the ability to resist smoking urges [52, 53]. Individuals with higher cognitive restraint may thus be better equipped to manage cravings. Moreover, the top ranked variable of Stroop performance (congruent condition) for distinguishing quitters from non‐quitters suggests specific cognitive control aspects may be more predictive than the classic Stroop interference effect [49, 54], and highlights the importance of discrete cognitive control processes in supporting successful smoking cessation [11, 38, 55].

In addition to these neurocognitive factors, sociodemographic‐environmental characteristics emerged as strong predictors. Consistent with established literature [14, 56], smoking within personal and occupational networks strongly distinguished smokers from never smokers. Aligning with prior studies [14, 57], we further identified employment status as a key predictor of smoking cessation success. This may reflect the stabilizing effects of routine, the presence of social support networks, or enhanced financial security associated with employment.

Another crucial factor, highlighted by its top ranking in the SHAP feature importance analysis, was alcohol use (as indicated by AUDIT scores), which likely reflects the well‐established comorbidity between alcohol and tobacco use [50, 51]. Higher alcohol consumption is often associated with persistent smoking and a lower likelihood of cessation. Notably, even moderate alcohol intake has been shown to impair P50 sensory gating [58], suggesting a neurobiological pathway through which alcohol may disrupt prefrontal function relevant to self‐regulation in the context of tobacco consumption. Our classifier may have captured this interaction between alcohol use and frontal P50 gating, reflecting the interrelationship of behavioural and neurocognitive factors impacting cognitive control and smoking behaviour.

Collectively, these findings underscore the complex interplay between cognitive, neurobiological, and environmental factors in shaping smoking behaviour and influencing long‐term cessation outcomes. While individual differences in PFC‐mediated cognitive control—such as decision‐making processes and responses to smoking cues—are critical, environmental factors exert substantial pressure and likely interact with neurobiological processes over time. While traditional risk factors, such as the degree of nicotine dependence, represent significant predictors of relapse, particularly in the early stages of cessation [59, 60], their predictive power for sustained abstinence can vary. Understanding the full scope of challenges in smoking cessation benefits from a multifaceted approach. This suggests that EEG could offer an added layer of understanding regarding the mechanisms of long‐term relapse and recovery, potentially identifying neurobiological markers of treatment success or relapse vulnerability that may not be fully captured by self‐report or behavioural measures alone. For instance, persistently reduced theta power in both current and long‐term former smokers may reflect a persistent impairment in cognitive control, potentially indicating a latent vulnerability that predisposes individuals to relapse despite prolonged abstinence [43].

These findings also demonstrate the potential of machine learning models that integrate multi‐domain data to advance personalized smoking interventions. By identifying individuals with specific risk profiles—such as reduced P50 gating, impaired cognitive control or high exposure to smoking in personal networks—these models can inform more targeted prevention and cessation strategies aimed at improving public health outcomes. For instance, individuals with marked neurophysiological vulnerabilities (e.g., significant P50 gamma deficits) might benefit from adjunctive neuromodulation approaches like NIBS targeting prefrontal circuits, while those in smoking‐conducive social environments may require focused behavioural or social network‐based interventions. When combined with standard treatments such as cognitive behavioural therapy (CBT), this precision‐guided approach could render the allocation of therapeutic resources more efficient and increase long‐term cessation rates. Ultimately, these results support incorporating multimodal predictive tools into clinical decision‐making to enable more effective, individualized prevention and cessation treatment strategies.

However, in interpreting these findings and pursuing clinical translation, several limitations of the current study warrant consideration. First, the representativeness of our cohort and the attrition observed over the 10‐year follow‐up period may impact the generalizability of long‐term predictions. Moreover, while the identified variables strongly predict smoking‐related behaviours, they do not establish a causal relationship with smoking. Additionally, the extended follow‐up duration may have introduced various unmeasured confounders that could influence the results. Moving forward, future research should prioritize longitudinal studies that integrate electrophysiology, neuropsychological assessments, neuroimaging and advanced machine learning models to capture the dynamic processes of nicotine dependence development and cessation. Translating these predictive insights into clinically viable tools represents an essential next step in advancing personalized interventions.

In conclusion, by integrating sociodemographic, electrophysiological, neuropsychological and clinical measures within a unified machine learning framework, our study captures the complex and enduring nature of tobacco use. Frontal functioning, cognitive control and social‐environmental factors emerged as key determinants across smoker phenotypes, highlighting the dynamic interplay between individual and environmental influences on tobacco behaviour. These findings underscore the central role of prefrontal mechanisms in nicotine addiction and demonstrate the potential of multimodal machine learning models for long‐term smoking behaviour prediction. In line with neuroimaging, NIBS and prior predictive modelling studies, our results further solidify the prefrontal cortex as a critical intervention target. By extending predictive modelling to a decade‐long follow‐up, our approach reveals the utility of multi‐domain models in risk stratification and personalized treatment. Future research should focus on translating these insights into actionable tools for guiding tailored prevention and cessation strategies in clinical practice.

## Author Contributions

**Pablo Reinhardt:** conceptualization, writing – original draft, data curation, software, methodology, formal analysis, visualization. **Norman Zacharias:** conceptualization, data curation, writing – review and editing. **Marinus Fislage:** conceptualization, writing – review and editing. **Justin Böhmer:** writing – review and editing. **Barbara Hollunder:** conceptualization, writing – review and editing. **Zala Reppmann:** writing – review and editing. **Anton Wiehe:** software, methodology. **Rebecca Rajwich:** data collection. **Nanne Dominick:** data collection. **Kerstin Ritter:** writing – review and editing. **Malek Bajbouj:** data curation. **Thomas Wienker:** data curation, writing – review and editing. **Jürgen Gallinat:** data curation, writing – review and editing. **Norbert Thürauf:** data curation, writing – review and editing. **Johannes Kornhuber:** data curation. **Falk Kiefer:** data curation, writing – review and editing. **Michael Wagner:** data curation, writing – review and editing. **Oliver Tüscher:** data curation, writing – review and editing. **Henrik Walter:** conceptualization, funding acquisition, writing – review and editing, resources, project administration. **Georg Winterer:** conceptualization, funding acquisition, writing – review and editing, resources, project administration.

## Ethics Statement

Data analysed in this study relies on study procedures that followed the 1975 Declaration of Helsinki, and informed consent was obtained from all participants before study participation. This study received ethical approval from the institutional review board of the Charité – Universitätsmedizin Berlin (vote EA2/031/19).

## Conflicts of Interest

The authors declare no conflicts of interest.

## Supporting information


**Table S1** Variables used in machine learning classification models.
**Table S2.** Model architecture of autoencoder.
**Table S3**. Classification performance of models (A)–(C).
**Table S4.** Prediction of quitters vs. non‐quitters using a pretrained model exclusively based on baseline data.
**Table S5.** Subsampling validation classification of (C) quitters versus non‐quitters.
**Table S6**. Replication with random forest classification and stacking for classification (C) quitters vs. non‐quitters.
**Table S7**. Sensitivity analysis: classification performance by domain.
**Figure S1.** Approximate feature importance of encoder model.
**Figure S2.** Area under the receiver operating curves (AUROC) across classification models.
**Figure S3.** Shapley (SHAP) feature importance plots of quitters vs. non‐quitters using a pretrained model and exclusively data of the baseline assessment.
**Figure S4.** Shapley (SHAP) feature importance plots of the subsampling validation classification of (C) quitters versus non‐quitters.
**Figure S5.** Shapley (SHAP) feature importance plots of classification quitters vs. non‐quitters leveraging random forest classification and stacking.

## Data Availability

The dataset analysed in this study is not publicly accessible due to data protection regulations but can be obtained from the corresponding author upon reasonable request. The source code used in this study is openly accessible and can be found in the following Zenodo GitHub repository: https://doi.org/10.5281/zenodo.15477769.
