# Supplementary material for: Machine Learning Classification of Smoking Behaviours—From Social Environment to the Prefrontal Cortex
Source: Addict Biol. 2025 Aug 6;30(8):e70056. doi: 10.1111/adb.70056 (PMC12328245; doi:10.1111/adb.70056)
Supplement: Supplementary file 1 — Table S1 Variables used in machine learning classification models. Table S2. Model architecture of autoencoder. Table S3. Classification performance of models (A)–(C). Table S4. Prediction of quitters vs. non‐quitters using a pretrained model exclusively based on baseline data. Table S5. Subsampling validation classification of (C) quitters versus non‐quitters. Table S6. Replication with random forest classification and stacking for classification (C) quitters vs. non‐quitters. Table S7. Sensitivity analysis: classification performance by domain. Figure S1. Approximate feature importance of encoder model. Figure S2. Area under the receiver operating curves (AUROC) across classification models. Figure S3. Shapley (SHAP) feature importance plots of quitters vs. non‐quitters using a pretrained model and exclusively data of the baseline assessment. Figure S4. Shapley (SHAP) feature importance plots of the subsampling validation classification of (C) quitters versus non‐quitters. Figure S5. Shapley (SHAP) feature importance plots of classification quitters vs. non‐quitters leveraging random forest classification and stacking. [file ADB-30-e70056-s001.docx]

**Supplementary Contents**

Reinhardt P, Zacharias N, Fislage M et al. Machine Learning Classification of Smoking Behavior – from social environment to prefrontal cortex.

**Table of Contents.**

**Supplementary Methods**

**Sample and Data Collection**

Description of the data collection and sampling.

**Machine Learning Classification**

Overall strategy and classification pipeline.

Additional information on baseline classification C) quitters vs. non-quitters.

Additional information replication of classification C) quitters vs. non-quitters.

Additional information on the autoencoder utilized for feature engineering in classification C) quitters vs. non-quitters.

**Supplementary Tables**

**Table S1**. Variables used in machine learning classification models.

**Table S2.** Model architecture of autoencoder.

**Table S3**. Classification performance of models A)-C).

**Table S4.** Prediction of quitters vs. non-quitters using a pretrained model exclusively based on baseline data.

**Table S5.** Subsampling validation classification of C) quitters vs. non-quitters**.**

**Table S6**. Replication with Random Forest Classification and Stacking for classification C) quitters vs. non-quitters.

**Table S7**. Sensitivity Analysis: classification performance by domain.

**Supplementary Figures**

**Fig. S1.** Approximate feature importance of encoder model.

**Fig. S2**. Area under the receiver operating curves (AUROC) across classification models.

**Fig. S3.** Shapley (SHAP) feature importance plots of quitters vs. non-quitters using a pretrained model and exclusively data of the baseline assessment

**Fig. S4.** Shapley (SHAP) feature importance plots of the subsampling validation classification of C) quitters vs. non-quitters

**Fig. S5.** Shapley (SHAP) feature importance plots of classification quitters vs. non-quitters leveraging Random Forest Classification and Stacking

**Supplementary Methods**

**Sample and Data Collection**

Data collection of the baseline German Nicotine Cohort study (NCS) took place between 2007-2009 in form of a nationwide population-based study with focus on current regular *smokers* and *never smokers*. The NCS cohort (N=2,396) is comprised of N = 1,116 current regular *smokers* vs. N=1,280 *never smokers and* features a standardized collection of a wide range of sociodemographic/environmental, clinical, neuropsychological, physiological, as well as electrophysiological parameters ^1–4^ (“Genetics of Nicotine Dependence and Neurobiological Phenotypes” – DFG priority program SPP1226). Study participants were randomly selected from the local general population via official resident registries in seven recruitment centers within Germany (Psychiatric University Hospital ambulances in Berlin, Bonn, Düsseldorf, Mannheim, Mainz, Aachen, and Erlangen). Among the 55,000 contacted subjects, 10% responded to the invitation via phone and agreed to participate. The most important exclusion criteria comprised being a former smoker or being affected by any type of neuropsychiatric disorders (including addiction) other than tobacco use disorder, among others.

Between 2019 to 2022, a subset of N=2,000 participants of the NCS baseline study from 2007-2009 were invited for a follow-up online survey via mail (in accordance with their prior consent for being re-contacted). Of the N=2,000 invited individuals, N=452 filled in the survey (response rate of 22.6%), of which N=311 were classified as *former* *never smokers* and N=141 as *former smokers* based on their smoking status at the time of the baseline assessment. Considering the current smoking status, within the group of former smokers, N=81 had continued smoking since (i.e., up until the time of completion of the follow-up survey) and were labeled as *non-quitters*. The remaining N=60 former smokers had been abstinent for at least one year before completing the survey and were thus labeled as *quitters*.

**Classification Models**

**Overall Strategy**

Our overall goal was to focus on comparability between applied classification pipelines (A)-C)). Therefore, we opted for homogeneous classification pipelines across classifications, with the extreme gradient boosting classifier (XGBClassifier) as a final classifier for all classifications. Also, we chose to use as few data transformation and variable preselection steps as possible to avoid overly complex models. We therefore relied on the onboard regularization of the XGBClassifier.

**Classification Pipeline**

Machine learning models were conducted by means of scikit-learn, version 0.24.2 and imbalanced-learn 0.8.1. Feature importance was conducted using the SHAP package (21.1.3). Hyperparameter optimization based on a randomized grid search with default arguments and the hyperparameter search space included the following parameters: maximum depth (4, 6, 8, 10), learning rate (0.01, 0.1, 0.2, 0.3), subsample (0.7, 0.8, 0.9, 1.0), gamma (0, 0.1, 0.3, 0.5, 1), and colsample by tree (0.6, 0.8, 1.0).

We used standard imputation by the most frequent value and the Isolation Forest (IF) algorithm for outlier detection (contamination level=’auto’ for analyses A) - B) and, due to the smaller sample size in analysis C), a contamination level of 0.1 as well as an oversampling group size of N=200 to boost the total sample size.

To further investigate feature importance, Shapley (SHAP) values were computed using the randomized grid search optimized classifier per cross-validation split and repetition. Bar plots of mean absolute SHAP values are the most straightforward method to visualize feature importance. In addition to this, bee swarm plots display SHAP explanations for each sample, where the x-axis position depends on the SHAP values of the samples. The density of samples (dots) piling up reflect the density of the feature. Moreover, the color of the dots (samples) represents the original raw value of the feature.

**Additional information on the prediction of quitters vs. non-quitters based only on baseline data and using a pretrained model**

To also investigate the stability of features found in classification C), we repeated this analysis, but restricted the input data to only baseline. As it had to be expected that predictive power would be limited, considering the time gap between baseline data collection and follow-up, we opted to use a pretrained XGB Classifier that was trained on classification of smokers vs. never-smokers (A) with almost identical settings to classification C), except we used SMOTE (instead of the Tomek variant) as well as n = 15 iterations (instead of n = 10) in the randomized grid search.

**Replications of classification model C)**

To confirm stability of the results of classification model C), we conducted a replication classification. Specifically, we repeated the classification with less missing electrophysiological sensory gating data. A random sampling approach was implemented in which for each of the ten repetitions, a subset of participants only with available electrophysiological sensory gating data was selected, thereby reducing the number of imputed EEG data. We used the built in pandas method sample with a fraction of 0.3, resulting in an N=131 (*quitters:* N=55 and *nonquitters:* N=76). SMOTETomek oversampling of both classes to N=100 was conducted.

**Additional information on classification analysis C)**

**Autoencoder model**

As the electrophysiological (EEG) domain comprised the most features of all domains, we aimed to exploit the baseline sample by using a deep autoencoder model for feature engineering (conducted with the functional API of TensorFlow 2.9.2). The architecture of the model was selected based on trial and error, although we noticed that there were no large performance differences between the different model architectures. The autoencoder was trained solely on Min Max scaled resting state and sensory gating derived EEG data. Of note, already processed EEG data was used. To avoid information leakage, the model was trained exclusively on those subjects’ EEG data who had not participated in the follow-up survey. For feature engineering, the encoder part of the autoencoder model was used, featuring five encoding (dense) layers using batch normalization, drop out (0.1) and sigmoid activation functions, with a bottleneck encoding layer of k=12. This model was trained with the Adam optimizer (learning rate: 0.0001) for 3000 epochs (shuffle=True) using binary cross entropy as loss function. The optimal architecture was determined by trial and error of different model parameters. For details on the final model architecture, please see Table S4. The mean weights of the input layer of the encoder as an approximation of feature importance is shown in Figure S1.

**Supplementary Tables**

**Table S1. Variables used in machine learning classification models.**

| **Sociodemographic and environmental variables** | | | | | | | |  |  |  |
| --- | --- | --- | --- | --- | --- | --- | --- | --- | --- | --- |
| Variable | Description | | | | | Item Options | |  |  |  |
| father_001 | Did your father ever smoke regularly? | | | |  | 1. Yes 2. No 3. Unknown | |  |  |  |
| father_002 | Did your father smoke in your shared living environment during your childhood? | | | |  | 1. Yes 2. No 3. Unknown | |  |  |  |
| father_003 | Does your father engage in smoking today? | | | |  | 1. Yes 2. No 3. Unknown | |  |  |  |
| mother_001 | Did your mother ever smoke regularly? | | | |  | 1. Yes 2. No 3. Unknown | |  |  |  |
| mother_004 | Did your mother smoke in your shared living environment during your childhood? | | | |  | 1. Omitted 2. Yes 3. No 4. Unknown | |  |  |  |
| siblings_001 | Do you have biological siblings? | | | |  | 1. Yes 2. No 3. Unknown | |  |  |  |
| siblings_002 | Did your sibling(s) ever smoke regularly? | | | |  | 1. Omitted 2. Yes 3. No 4. Unknown | |  |  |  |
| siblings_003 | Is one of your siblings a non-smoker? | | | |  | 1. Omitted 2. Yes 3. No 4. Unknown | |  |  |  |
| environment_001 | Have people regularly engaged in smoking in your professional environment? | | | |  | 1. Yes 2. No 3. Unknown | |  |  |  |
| environment_002 | If yes, do people still regularly engage in smoking in your professional environment? | | | |  | 1. Omitted 2. Yes 3. No 4. Unknown | |  |  |  |
| environment_003 | Have people still engaged in smoking in your private environment? | | | |  | 1. Omitted 2. Yes 3. No 4. Unknown | |  |  |  |
| environment_004 | If yes, do people still regularly engage in smoking in your private environment? | | | |  | 1. Omitted 2. Yes 3. No 4. Unknown | |  |  |  |
| school_001 | How many years did you go to school? | | | |  | Open text field | |  |  |  |
| profeession_001 | How many years were you in vocational training?  (Primary and secondary school certificate: max. 10 years, vocational diploma: max. 12 years, high school diploma: max. 13 years) | | | |  | Open text field | |  |  |  |
| profession_002 | What is your current professional situation? | | | |  | 1. Employed full-time 2. employed part-time 3. unemployed 4. unable to work 5. housewife/husband 6. student 7. pensioner 8. unknown | |  |  |  |
| profession_003 | What is the approximate annual income of your household? | | | |  | 1. Less than 10.000 2. 10.000- 20.000 3. 20.000-30.000 4. 30.000-40.000 5. 40.000-50.000 6. 50.000-60.000 7. more than 60.000 8. Unknown | |  |  |  |
| family_001 | Are you an adopted child? | | | |  | 1. Yes 2. No 3. Unknown | |  |  |  |
| family_002 | With whom did you grow up?  predominantly both biological parents, predominantly biological father, predominantly biological mother, predominantly both foster parents, predominantly foster father, predominantly foster mother, predominantly other, Unknown | | | |  | 1. predominantly both biological parents 2. predominantly biological father 3. predominantly biological mother predominantly both foster parents 4. predominantly foster father 5. predominantly foster mother predominantly other 6. Unknown | |  |  |  |
| family_003 | What is your current marital status? | | | |  | 1. Single 2. Married 3. Divorced 4. Separated 5. Widowed 6. Unknown | |  |  |  |
| family_004 | Do you live together with someone? | | | |  | 1. Yes 2. No 3. Unknown | |  |  |  |
| family_005 | Do you have own (biological) children? | | | |  | 1. Yes 2. No 3. Unknown | |  |  |  |
| family_006 | Has there been a history of psychiatric disorders in your family? | | | |  | 1. Yes 2. No 3. Unknown | |  |  |  |
| age |  | | | |  | Numeric | |  |  |  |
| gender |  | | | |  | Numeric | |  |  |  |
| **Clinical and neuropsychological variables** | | | | | | | |  |  |  |
| Test | Domain | | | | Variable definition | | |  |  |  |
| Block span  forward and backward of the Wechsler memory scale—revised (WMS-R) | visuo-spatial short-term working memory | | | |  | | |  |  |  |
|  |  | |  | | BLS_01 | | repeat numbers forward |  |  |  |
|  |  | |  | | BLS_02 | | repeat numbers backwards |  |  |  |
|  |  | |  | | bzt_001 | | number of rows reproduced correctly |  |  |  |
| Stroop task | Color-word interference test | | Inhibition / selective attention, Bäumler 1985) | |  | | |  |  |  |
|  |  | |  | | fwit_001 | | time needed for reading colored word items |  |  |  |
|  |  | |  | | fwit_002 | | Time needed for naming-colored strikes |  |  |  |
|  |  | |  | | fwit_003 | | time needed for interference attempt |  |  |  |
|  |  | |  | | fwit_004 | | errors interference attempts |  |  |  |
| Trail making test (TMT) | visual perception and task switching abilities | | | |  | | |  |  |  |
|  |  | |  | | tmt_001 | | time needed to complete for test part A (Testform-A) |  |  |  |
|  |  | |  | | tmt_002 | | time needed to complete test part B (Testform-B) |  |  |  |
|  |  | |  | |  | |  |  |  |  |
| Continuous Performance Test  (CPT) | selective attention, sustained attention, impulsive behavior | | | |  | | |  |  |  |
|  |  | |  | | cpt_001 | | proportion of hits |  |  |  |
|  |  | |  | | cpt_002 | | proportion false alarms |  |  |  |
|  |  | |  | | cpt_003 | | proportion randoms |  |  |  |
|  |  | |  | | cpt_004 | | reaction time (hits) |  |  |  |
|  |  | |  | | cpt_005 | | variance reaction time (hits) |  |  |  |
|  |  | |  | | cpt_006 | | Deprime |  |  |  |
|  |  | |  | | cpt_007 | | Log Beta |  |  |  |
|  |  | |  | |  | |  |  |  |  |
| Verbal learning and memory test  VLMT | Trials 1–6 (learning and  immediate recall | | | |  | | |  |  |  |
|  |  | |  | | vlm_001 | | number correct words after first learning pass |  |  |  |
|  |  | |  | | vlm_002 | | number correct words after fifth learning pass |  |  |  |
|  |  | |  | | vlm_003 | | sum of correct words over all five passes |  |  |  |
|  |  | |  | | vlm_004 | | number of correct words after interference list A6 |  |  |  |
|  |  | |  | | vlm_005 | | number correct words with delayed reproduction A7 |  |  |  |
|  |  | |  | | vlm_006 | | number of correct words of list A (recognition task) |  |  |  |
| digit symbol substitution test (WAIS-R) | repeat numbers | |  | | zsn_001 | | number of correct rows of forward repeating numbers |  |  |  |
|  |  | |  | | zsn_002 | | number correct rows of backward repeating numbers |  |  |  |
| Verbal Fluency Letter (VFL) test |  | |  | | vfl_001 | | number correct reproduced words |  |  |  |
|  |  | |  | | vfl_002 | | number repitions/errors |  |  |  |
|  |  | |  | | vfl_003 | | number correct produced words |  |  |  |
|  |  | |  | | vfl_004 | | number of repitions/errors |  |  |  |
|  |  | |  | | vfl_005 | | number correct reproduced words |  |  |  |
|  |  | |  | | vfl_006 | | number repitions/errors |  |  |  |
|  |  | |  | | vfl_007 | | number correct produced words |  |  |  |
|  |  | |  | | vfl_008 | | number repitions/errors |  |  |  |
|  |  | |  | | vlm_007 | | correct recognition performance |  |  |  |
|  |  | |  | | vlm_008 | | wrong recognized of list B |  |  |  |
| multiple choice vocabulary test MWT-B |  | |  | | mwt_001 | | number of correct words |  |  |  |
| NEO FFI 5 |  | |  | | neo_001_e | | neuroticism |  |  |  |
|  |  | |  | | neo_002_e | | extraversion |  |  |  |
|  |  | |  | | neo_003_e | | openness |  |  |  |
|  |  | |  | | neo_004_e | | agreeableness |  |  |  |
|  |  | |  | | neo_005_e | | consciousness |  |  |  |
| Tridimensional Personality Questionnaire (TPQ) |  | |  | |  | |  |  |  |  |
|  |  | |  | | tpq_001 | | spontaineity |  |  |  |
|  |  | |  | | tpq_002 | | risk aversion |  |  |  |
|  |  | |  | | tpq_003 | | reward dependency |  |  |  |
| Questionnaire of Eating Behavior  (FEV) |  | |  | | fev_001 | | Cognitive control of eating behavior |  |  |  |
|  |  | |  | | fev_002 | | disorder of eating behavior |  |  |  |
|  |  | |  | | fev_003 | | feelings of hunger |  |  |  |
|  |  | |  | | onset_001 | | How old were you when you started with occasional or habitual smoking? |  |  |  |
|  |  | |  | | onset_002 | | How many cigarettes did you smoke per day? |  |  |  |
| Pittsburgh Sleep Quality Index (PSQI) |  | |  | | psqi_008 | | overall value |  |  |  |
| State and Trait Inventory (STAI) |  | |  | |  | |  |  |  |  |
|  |  | |  | | stai_001 | | X1 |  |  |  |
|  |  | |  | | stai_002 | | X2 |  |  |  |
| Packyears* |  | |  | |  | | calculated as number of cigarette packs x number of years as a smoker |  |  |  |
| Daily smoking* |  | |  | | z_day | | Daily cigarettes smoked |  |  |  |
| onset_001* |  | |  | | onset_001 | | How old were you when you began smoking (occasionally or regularly?) |  |  |  |
| onset_002* |  | |  | | onset_002 | | How many cigarettes did you then smoke? |  |  |  |
|  |  | |  | |  | |  |  |  |  |
| Mean_diff_phases* | | |  | |  | | Subjects were asked if they had tried quitting smoking, and if so, for how long. For the first four phases, the mean (duration of quitting) was computed. |  |  |  |
| Fagerström test for nicotine dependence* (FTND) | sum score FTND | |  | |  | | ftnd10 |  |  |  |
| Questionnaire of Smoking Urges* (QSU) | sum score QSU | |  | |  | | qsu |  |  |  |
| Alcohol Use Disorders Identification Test (AUDIT) | sum score AUDIT | |  | |  | | audit_001 |  |  |  |
| Perceived Stress Scale (PSS) |  | |  | |  | | sum score |  |  |  |
| ADHD-Checklist |  | |  | |  | | adhd_001 |  |  |  |
| Beck Depression Inventory (BDI) | sum score BDI | |  | |  | | bdi_001 |  |  |  |
| Zahlen-Symbol Test (Kaplan  et al. 1991) | Digit-Symbol Test | |  | |  | | zst_001 |  |  |  |
| blood pressure |  | |  | |  | |  |  |  |  |
|  |  | |  | |  | | c_rrs |  |  |  |
|  |  | |  | |  | | c_rrd |  |  |  |
| handedness |  | |  | |  | | c_haendig |  |  |  |
| d_psychiatric |  | |  | |  | | Were or are psychiatric disorders present? |  |  |  |
| Body Mass Index (BMI) |  | | | | | |  |  |  |  |
| ***Electrophysiological variables*** | | | | | | | |  |  |  |
| Power Spectral Density (PSD) Electrodes (Resting State) | | | | | | | |  |  |  |
| Left | Right |  | |  | | | |  |  |  |
| Fp1 | Fp2 |  | |  | | | |  |  |  |
| F3 | F4 |  | |  | | | |  |  |  |
| F7 | F8 |  | |  | | | |  |  |  |
| A1 | A2 |  | |  | | | |  |  |  |
| T3 | T4 |  | |  | | | |  |  |  |
| C3 | C4 |  | |  | | | |  |  |  |
| T5 | T6 |  | |  | | | |  |  |  |
| P3 | P4 |  | |  | | | |  |  |  |
| O1 | O4 |  | |  | | | |  |  |  |
| - | - | Fz | |  | | | |  |  |  |
| - | - | Cz | |  | | | |  |  |  |
| - | - | Pz | |  | | | |  |  |  |
| Sensory Gating Paradigm | | | | | | | |  |  |  |
| For the sensory gating paradigm, participants were instructed to passively listen to N=100 binaurally presented pairs of two 250 sinus tones (2,000 Hertz; duration of 50 milliseconds; fixed interstimulus interval of 500 milliseconds between the first (S1) and second (S2) sinus tone; pseudo randomization of intervals (5 to 9 seconds, mean of 7 seconds)). P50 responses were defined as the maximum positive peak (48 and 68 milliseconds after onset of both S1 and S2). The amplitudes of P50 were measured based on the preceding negativity (S1 mean latency: 56 ± 4 milliseconds; S2 mean latency: 57 ± 5 milliseconds at electrode position Cz).  Lower values of the difference indicate either weak inhibition of the second tone (no gating or filtering), or low S1 onset related P50 amplitudes.  For the analysis of time-frequency, a discrete Daubechies wavelet transform was applied. Voltage bands were based on 48 to 74 milliseconds time windows after S1 and S2 across frequency bands (see below). | | | | | | | |  |  |  |
| P50 Fz Event Related Potential | | | | q_Fz_P50 | | | |  |  |  |
| P50 Cz Event Related Potential | | | | q_Cz_P50 | | | |  |  |  |
| P50 Pz Event Related Potential | | | | q_Pz_P50 | | | |  |  |  |
| Daubechies wavelets alpha resolution components (16-32 Hz) | | | |  | | | |  |  |  |
|  | | | | q_Fz_alpha | | | |  |  |  |
|  | | | | q_Cz_alpha | | | |  |  |  |
|  | | | | q_Pz_alpha | | | |  |  |  |
|  | | | |  | | | |  |  |  |
| Daubechies wavelets gamma resolution components (32-60Hz) | | | |  | | | |  |  |  |
|  | | | | q_Fz_gamma | | | |  |  |  |
|  | | | | q_Cz_gamma | | | |  |  |  |
|  | | | | q_Pz_gamma | | | |  |  |  |
| Daubechies wavelets theta resolution components (8-16Hz) | | | |  | | | |  |  |  |
|  | | | | q_Fz_theta | | | |  |  |  |
|  | | | | q_Cz_theta | | | |  |  |  |
|  | | | | q_Pz_theta | | | |  |  |  |
| **Follow-Up Questionnaire** | | | | | | | |  |  |  |
| variable description |  | |  | |  | | Item answer option |  |  |  |
| b05_soz_03  marital status | Please tell us your marital status | | | | | | 1. single 2. married or in a relationship 3. separated 4. divorced |  |  |  |
| b05_soz_04  parenthood | Do you have own kids? | | | | | | 1. Yes 2. No |  |  |  |
|  |  | |  | |  | |  |  |  |  |
| b05_soz_05  size of residence | What is the size of your place of residence (number of inhabitants)? | | | | | | 1. up to 5000 2. up to 20000 3. up to 50000 4. up to 100000 5. up to 500000 6. above 500000 |  |  |  |
| b05_soz_06 | In the last 3 months, have you been full employed? | | | | | | 1. Yes 2. No |  |  |  |
| b05_soz_07  monthly income | What is your personal average monthly net income? | | | | | | 1. below 500€ 2. 500 – 1000€ 3. 1000 – 1500€ 4. 1500 – 2000€ 5. 2000 – 2500€ 6. 2500 – 3000€ 7. 3500 – 4000€ 8. 4000 – 4500€ 9. 4500 – 5000€ 10. above 5000€ |  |  |  |
| b05_soz_08  living alone | Do you live alone? | | | | | | 1. yes 2. no |  |  |  |
| b05_soz_09  living alone | Do you take drugs? | | | | | | 1. yes   no |  |  |  |
| b05_soz_10  mental health | Have you ever had professional help due to mental problems? | | | | | | 1. yes 2. no |  |  |  |
| b05_soz_11  mental health | Are you currently being treated by a psychiatrist? | | | | | | 1. yes 2. no |  |  |  |
| b05_soz_12  Physical activity per week | We would like to know in what way and how often you are physically active in everyday life. How often do you practice a strenuous physical exercise in everyday life?  activity, for example in almost never or never sport, during heavy work at home or at work? | | | | | | 1. more than once a week 2. once a week 3. one to three times a month 4. almost never |  |  |  |
| b05_soz_13  body weight | Body weight (approximate) | | | | | | Open text field |  | open text field |  |
| b05_soz_14  physical health | Since your last study participation, did you have a serious illness? | | | | | | Open text field |  |  |  |
| Perceived Stress Scale (PSS) |  | | | | | | Sum score |  | psqi_008 | overall value |
| Beck Depression Inventory (BDI) |  | | | | | | Sum score |  |  |  |
| Alcohol Use Disorders Identification Test (AUDIT) |  | | | | | | Sum score |  |  |  |

*Notes.* *Smoking related variables marked with an asterisk were solely used in the longitudinal classification model of quitters versus non-quitters.

**Table S2. Model architecture used for training of the autoencoder.**

| **Layer (type)** | **Output Shape** | **Param #** |
| --- | --- | --- |
| input_2 (Input Layer) | [(None, 234)] | 0 |
| dense_8 (Dense) | (None, 468) | 109980 |
| dropout_6 (Dropout) | (None, 468) | 0 |
| batch_normalization_7 | (None, 468) | 1872 |
| dense_9 (Dense) | (None, 234) | 109746 |
| dropout_7 (Dropout) | (None, 234) | 0 |
| batch_normalization_9 | (None, 234) | 936 |
| dense_11 (Dense) | (None, 58) | 13630 |
| dense_12 (Dense) | (None, 12) | 708 |
| batch_normalization_11 | (None, 12) | 48 |
| dropout_10 (Dropout) | (None, 12) | 0 |
| batch_normalization_12 | (None, 12) | 48 |
| dense_14 (Dense) | (None, 117) | 1521 |
| dropout_11 (Dropout) | (None, 117) | 0 |
| batch_normalization_13 | (None, 117) | 468 |
| dense_15 (Dense) | (None, 234) | 27612 |
|  |  |  |
| Total params 266,569  Trainable params 264,883  Non-trainable params 1,686 | | |

**Table S3. Results of classification models.**

| \| Classification Precision Recall F1-Score Accuracy AUROC (mean ± std) \| \| \| \| \| \| --- \| --- \| --- \| --- \| --- \| \| A) Smokers vs. Never Smokers \| \| \| \| \| \| 0.78 0.86 (± 0.03) \| \| \| \| \| \| Never Smokers \| 0.79 \| 0.81 \| 0.80 \|  \| \| Smokers \| 0.76 \| 0.73 \| 0.75 \|  \| \| B) Heavy Smokers vs. Never Smokers \| \| \| \| \| \| 0.89 0.92(± 0.03) \| \| \| \| \| \| Heavy Smokers \| 0.78 \| 0.63 \| 0.69 \|  \| \| Never Smokers \| 0.91 \| 0.95 \| 0.93 \|  \| \| C) Quitters vs. Non-Quitters \| \| \| \| \| \| 0.65 0.69 (± 0.13) \| \| \| \| \| \| Quitters \| 0.59 \| 0.50 \| 0.54 \|  \| \| Non-Quitters \| 0.67 \| 0.74 \| 0.70 \|  \| \| Replication of C) \|  \|  \| 0.67 0.71 (± 0.13) \| \| \| Quitters \| 0.70 \| 0.79 \| 0.74 \|  \| \| Non-Quitters \| 0.58 \| 0.46 \| 0.52 \|  \| \|  \| \| \| \| \| |
| --- | --- | --- | --- | --- | --- | --- | --- | --- | --- | --- | --- | --- | --- | --- | --- | --- | --- | --- | --- | --- | --- | --- | --- | --- | --- | --- | --- | --- | --- | --- | --- | --- | --- | --- | --- | --- | --- | --- | --- | --- | --- | --- | --- | --- | --- | --- | --- | --- | --- | --- | --- | --- | --- | --- | --- | --- | --- | --- | --- | --- | --- | --- | --- | --- | --- | --- | --- | --- | --- | --- | --- | --- | --- | --- | --- | --- | --- | --- | --- | --- | --- | --- | --- | --- | --- |

*Abbreviations:* AUROC, area under the receiver operating curve; EEG, electroencephalography; SD, standard deviation.

**Table S4.** **Prediction of quitters vs. non-quitters using a pretrained model using only baseline data**

|  | Precision | Recall | F1 score |  |
| --- | --- | --- | --- | --- |
| Quitters | 0.49 | 0.40 | 0.44 |  |
| Non-Quitters | 0.61 | 0.69 | 0.65 |  |
|  |  |  |  |  |
|  | Weighted average |  |  | mean ± std |
| Weighted average | Precision | Recall | F1 score | AUROC |
| Quitters vs. Non-Quitters | 0.56 | 0.57 | 0.56 | 0.58 (± 0.14) |

*Abbreviations:* AUROC, area under the receiver operating curve; std, standard deviation. Top:

precision, recall and f 1 score for each class separated. Bottom: Weighted averages of the same measures (precision, recall, f1 score) and additionally the mean AUROC score.

**Table S4. Additional information on classification success of subsampling validation classification of quitters vs. non-quitters**

|  | Weighted average | | | mean ± std |
| --- | --- | --- | --- | --- |
|  | Precision | Recall | F1 score | AUROC |
| Quitters vs. Non-Quitters | 0.66 | 0.67 | 0.67 | 0.73 (± 0.14) |

*Abbreviations:* AUROC, area under the receiver operating curve; std, standard deviation.

**Table S5. Additional robustness check for classification of quitters vs. non-quitters – replication with Random Forest Classification and Stacking.**

|  | Weighted average | | | mean ± std |
| --- | --- | --- | --- | --- |
|  | Precision | Recall | F1 score | AUROC |
| Random Forest | 0.6 | 0.58 | 0.57 | 0.63 (± 0.14) |
| Stacking | 0.59 | 0.59 | 0.58 | 0.65 (± 0.14) |

*Abbreviations:* AUROC, area under the receiver operating curve; std, standard deviation.

**Table S7.** Classification performance by domain.

| AUROC (M±SD) | | | | |
| --- | --- | --- | --- | --- |
|  | Sociodemographic  environmental | Clinical | Neuropsychological | EEG |
| Quitters vs.  Nonquitters | 0.63 (± 0.15) | 0.6 (± 0.14) | 0.61 (± 0.14) | 0.51 (± 0.15) |
| Smokers vs.  Never Smokers | 0.78 (± 0.04) | 0.68 (± 0.03) | 0.62 (± 0.04) | 0.7 (± 0.05) |
| Heavy Smokers vs.  Never Smokers | 0.84 (± 0.05) | 0.71 (± 0.05) | 0.7 (± 0.06) | 0.83 (± 0.04) |

*Abbreviations:* AUROC, area under the receiver operating curve; EEG, electroencephalography; M, mean; SD, standard deviation.

**Supplementary Figures**


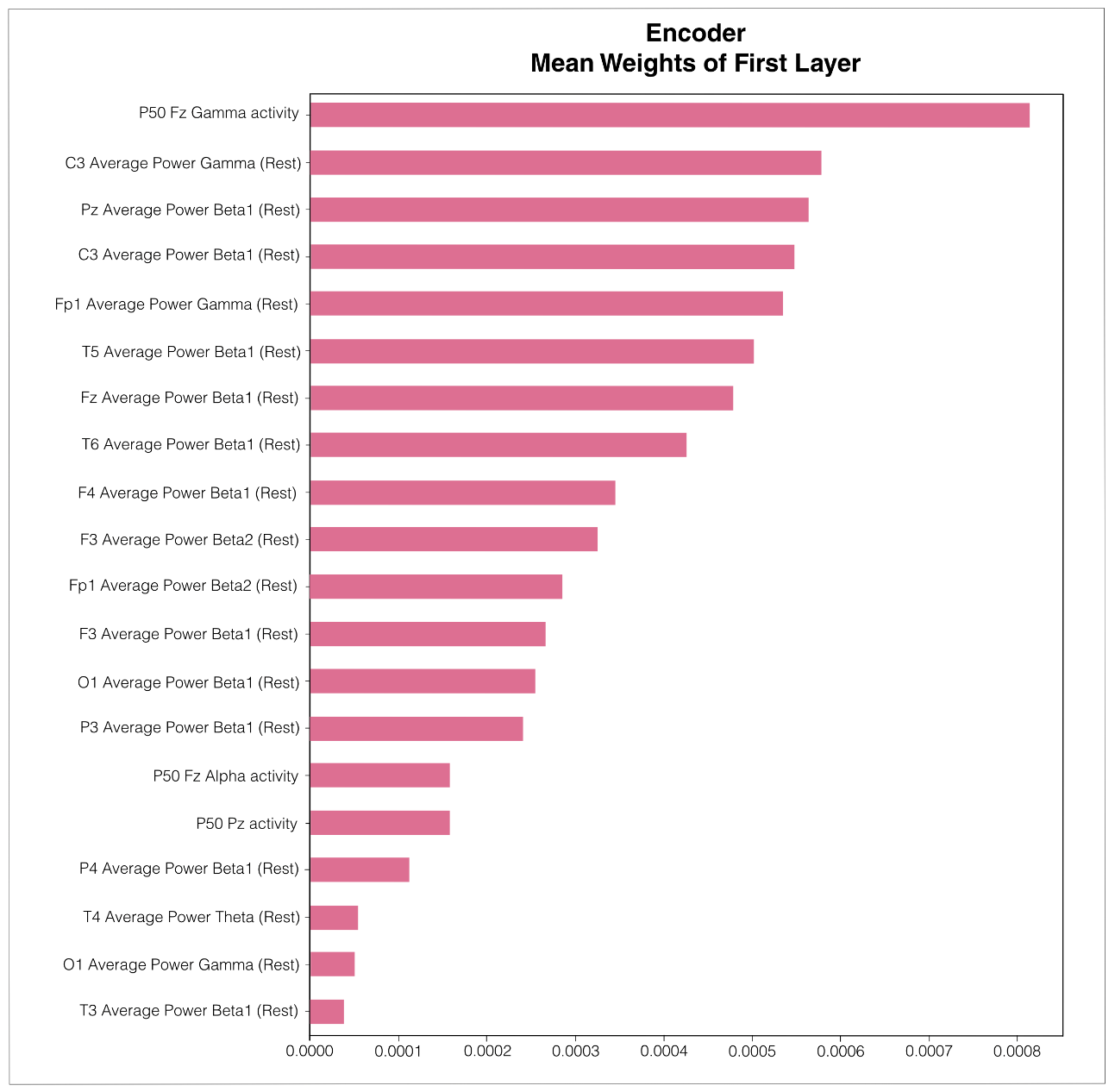


**Fig. S1.** **Approximate feature importance of encoder model.** Mean weights (input layer) of the first twenty most highly weighted features are displayed, sorted in descending order.


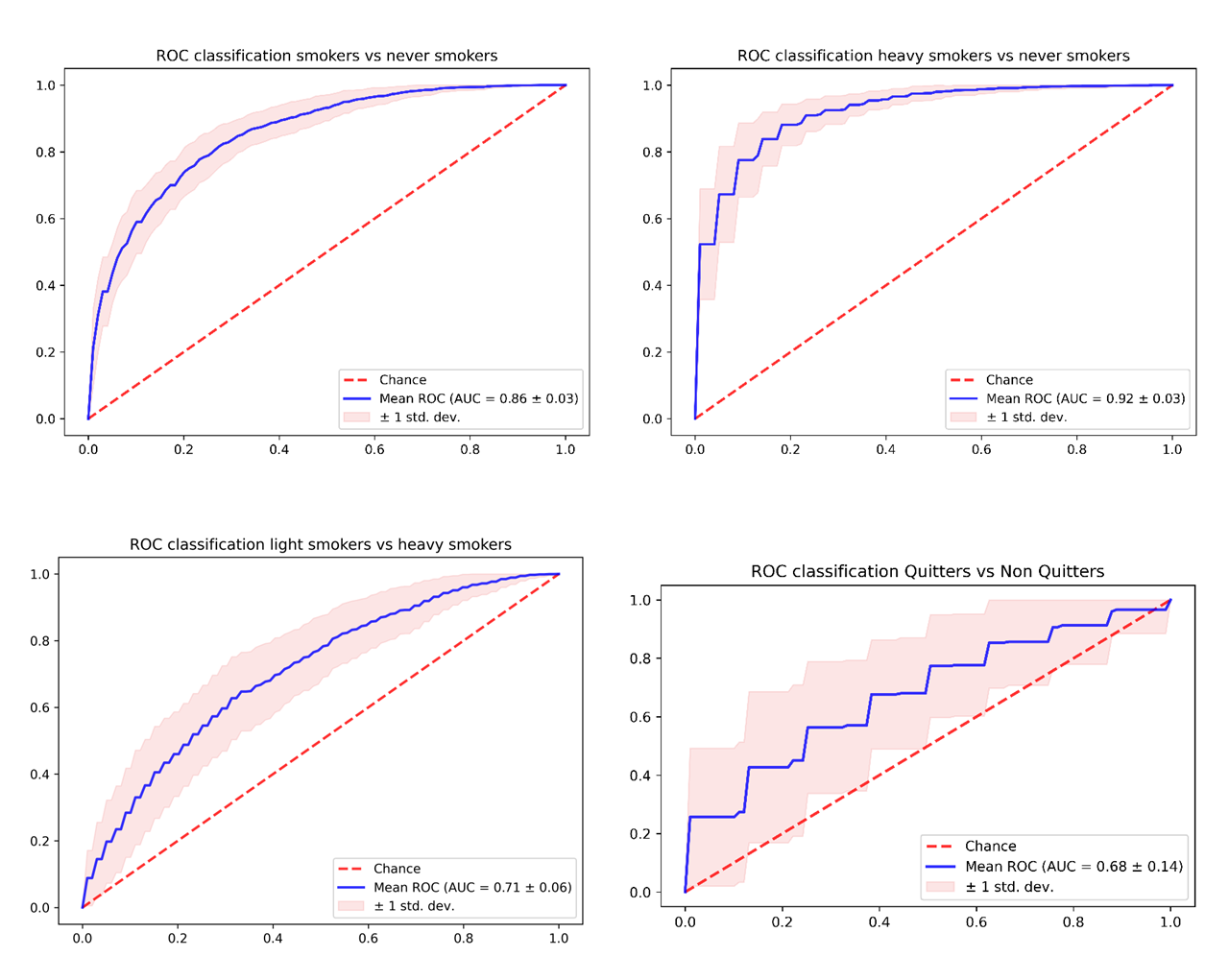


**Fig. S2. Area under the receiver operating curves (AUROC) across classification models.** *Top row from left to right:* Smokers vs. never-smokers; heavy smokers vs. never-smokers. *Bottom row from left to right:* Light smokers vs. heavy smokers, quitters vs. non-quitters. *Abbreviations:* AUC, area under the curve; ROC, receiver operating curve; std. dev., standard deviation.


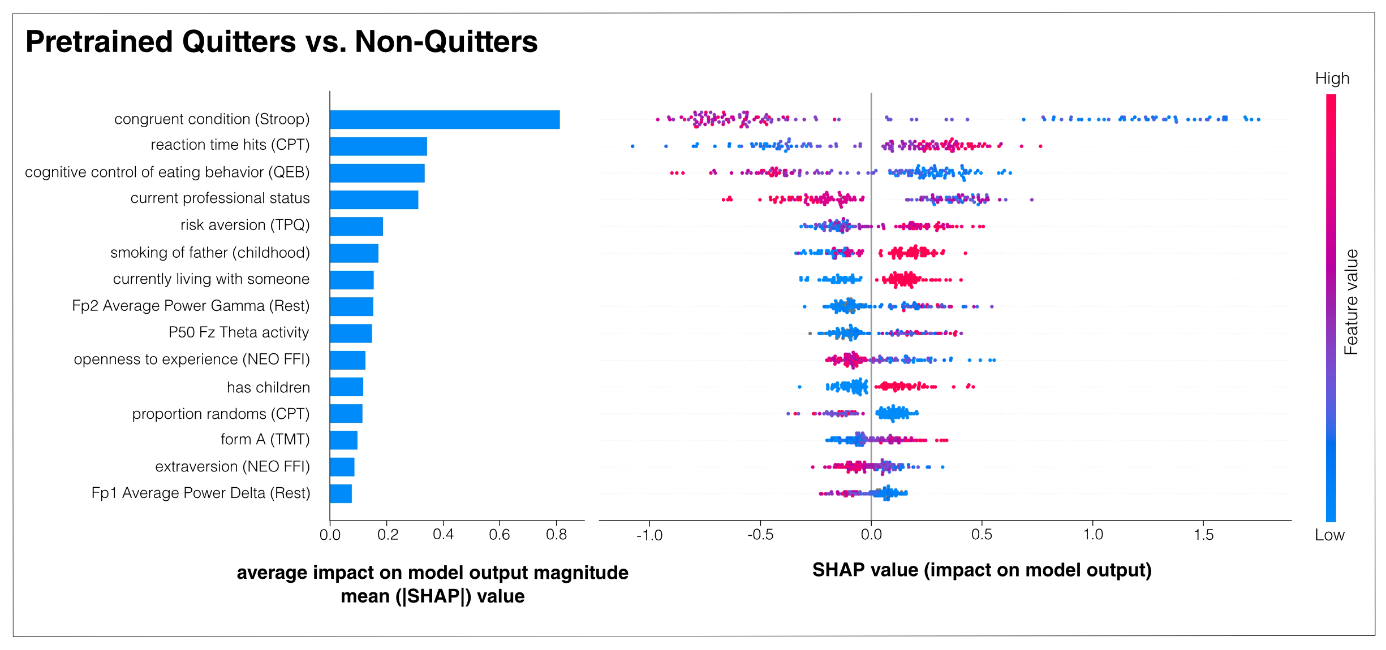


**Fig. S3. Shapley (SHAP) feature importance plots of quitters versus non-quitters based solely on baseline data, using a pretrained classifier.** *Left:* The bar plot displays the top fifteen most important features in descending order. *Right:* The same information is shown in the right plot. Additionally, positive SHAP values mark the contribution of the positive class (quitters). Negative SHAP values indicate feature contribution in favor of the corresponding negative class (non-quitters). Larger absolute SHAP values relate to a larger impact on model output. *Abbreviations*: congruent condition (Stroop) = first Stroop task condition, CPT = Continuous Performance Test, QEB = Questionnaire of eating behavior, TPQ = Tridimensional Personality Questionnaire, NEO FFI = NEO Five-Factor Inventory, TMT = Trail Making Test.


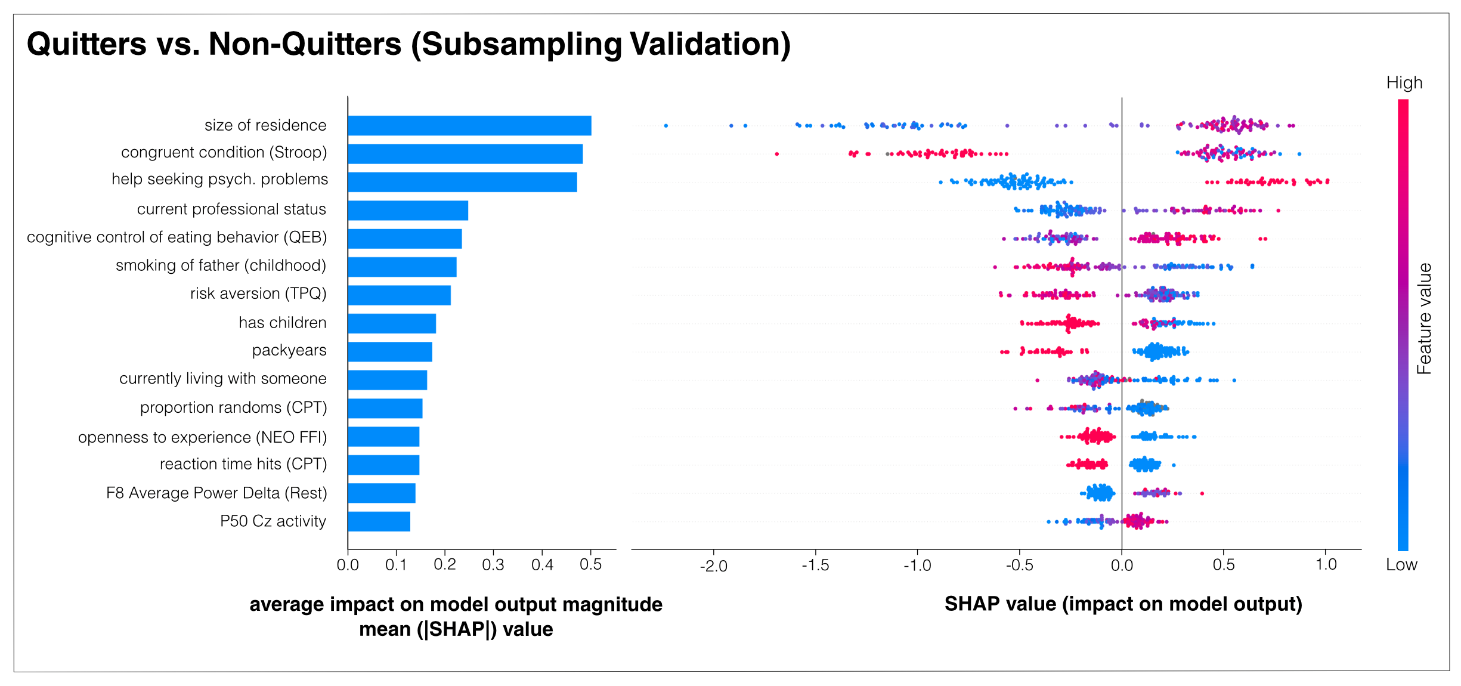


**Fig. S4. Shapley (SHAP) feature importance plots of the subsampling validation of the classification of quitters versus non-quitters (model C)**. *Left:* The bar plot displays the top fifteen most important features in descending order. *Right:* The same information is shown in the right plot. Additionally, positive SHAP values mark the contribution of the positive class (quitters). Negative SHAP values indicate feature contribution in favor of the corresponding negative class (non-quitters). Larger absolute SHAP values relate to a larger impact on model output. *Abbreviations*: congruent condition (Stroop) = first Stroop task condition, QEB = Questionnaire of eating behavior, CPT = Continuous Performance Test, TPQ = Tridimensional Personality Questionnaire, NEO FFI = NEO Five-Factor Inventory.


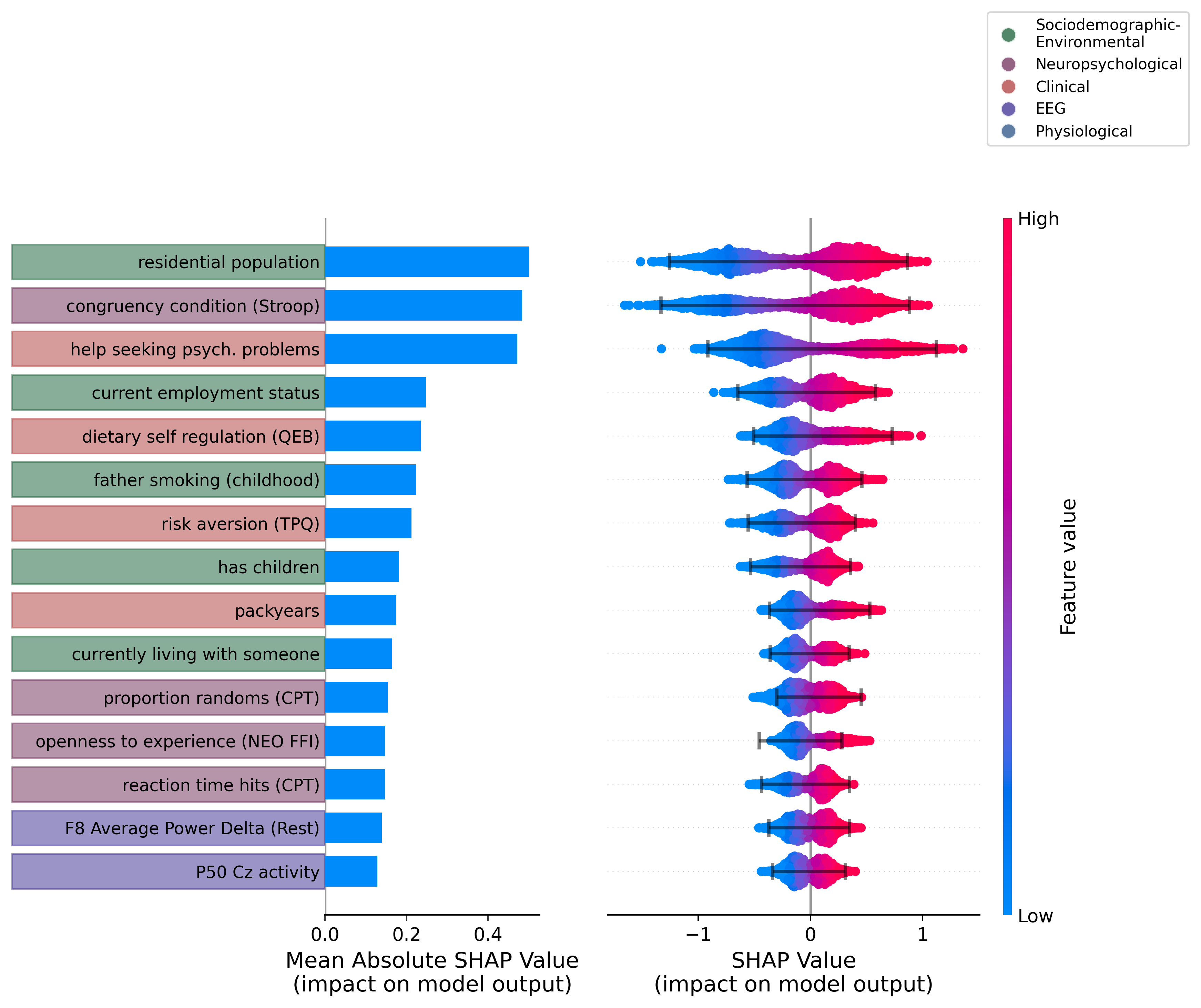

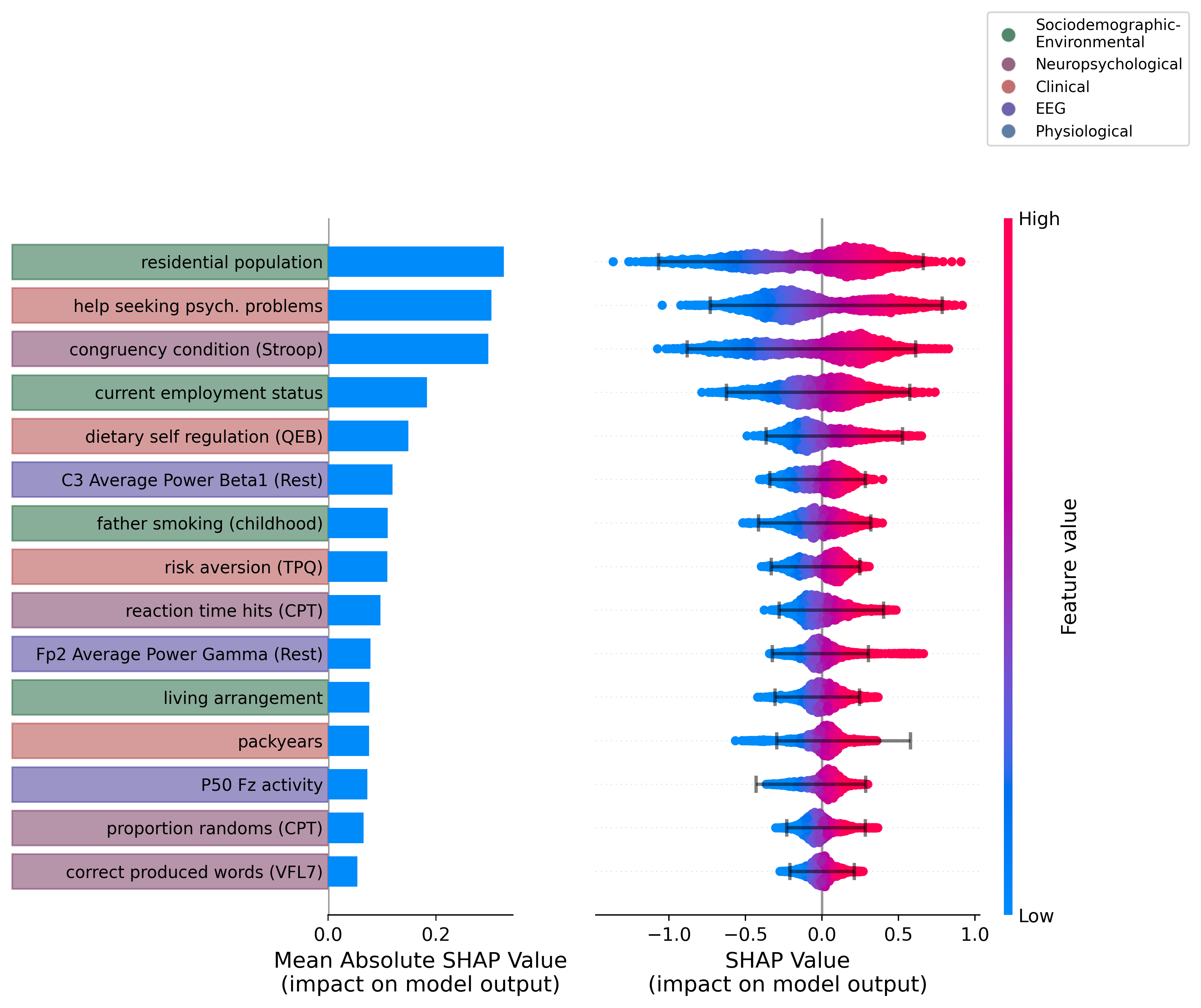


**Fig. S5.** **Shapley (SHAP) feature importance plots for the classification of Quitters vs. Non-Quitters for Replications conducted Random Forest Classifier and Stacking**. *Left*: displays the Random Forest Classification model. The bar plot displays the top fifteen most important features in descending order for the Random Forest Classification Model. The same information is shown in the right plot, where positive SHAP values mark the contribution of the positive class. *Right:* Stacking Classification of Quitters vs. Non-Quitters. The interpretation of the bar- and beeswarm plot is identical to the description of the left side.

**REFERENCES**

1. Lindenberg A, Brinkmeyer J, Dahmen N, et al. The German multi-centre study on smoking-related behavior-description of a population-based case-control study: The German multi-centre study on smoking-related behavior. *Addiction Biology*. 2011;16(4):638-653. doi:10.1111/j.1369-1600.2011.00322.x

2. Brinkmeyer J, Mobascher A, Musso F, et al. P50 sensory gating and smoking in the general population: P50 sensory gating & smoking. *Addiction Biology*. 2011;16(3):485-498. doi:10.1111/j.1369-1600.2010.00302.x

3. Cohrs S, Rodenbeck A, Riemann D, et al. Impaired sleep quality and sleep duration in smokers-results from the German Multicenter Study on Nicotine Dependence: Sleep in smokers. *Addiction Biology*. 2014;19(3):486-496. doi:10.1111/j.1369-1600.2012.00487.x

4. Wagner M, Schulze-Rauschenbach S, Petrovsky N, et al. Neurocognitive impairments in non-deprived smokers-results from a population-based multi-center study on smoking-related behavior: Cognition in smokers. *Addiction Biology*. 2013;18(4):752-761. doi:10.1111/j.1369-1600.2011.00429.x
